# Attacking *Plasmodium vivax*

**DOI:** 10.4269/ajtmh.16-0517

**Published:** 2016-12-28

**Authors:** J. Kevin Baird

**Affiliations:** 1 Eijkman-Oxford Clinical Research Unit, Jakarta, Indonesia.; 2 Centre for Tropical Medicine, Nuffield Department of Medicine, University of Oxford, Oxford, United Kingdom.

## Abstract

Discussions beginning in 2012 ultimately led to a landmark document from the World Health Organization (WHO) titled, *Control and Elimination of Plasmodium vivax: A Technical Brief*, published in July 2015. That body of work represents multiple expert consultations coordinated by the WHO Global Malaria Program, along with technical consensus gathering from national malaria control programs via the WHO regional offices around the globe. That document thus represents thoroughly vetted state-of-the-art recommendations for dealing specifically with *P. vivax*, the first assembly of such by the WHO. This supplement to the journal was commissioned by the WHO and compiles the very substantial body of evidence and analysis informing those recommendations. This introductory narrative to the supplement provides the historical and technological context of global strategy for combatting *P. vivax* and reducing the burdens of morbidity and mortality it imposes.

## Pernicious Malaria

The clinical condition with infection by *Plasmodium vivax* worsens perniciously, more slowly and less dramatically than with infection by *Plasmodium falciparum*. The seemingly sluggish progress of the infection and its comparatively low levels of parasitemia lulled us into thinking it an intrinsically benign species. That false aegis fostered a deep neglect of *P. vivax* in research, clinical medicine, and public health throughout the second half of the 20th century. Today, we understand that poor access to limited health care results in delayed or inadequate therapy, and in such settings, *P. vivax* malaria often progresses to states of severe illness essentially similar to those of *P. falciparum* malaria. In the impoverished and often isolated rural tropics, endemic *P. vivax* threatens patients and populations because serious problems in the diagnosis, treatment, and control of that infection have not been solved. However, the long deferral of action in doing so may be considered nearly ended.

This extraordinary supplement of the *American Journal of Tropical Medicine and Hygiene* contains much of the evidence underpinning the World Health Organization's (WHO) *Control and Elimination of Plasmodium vivax: A Technical Brief*^1^ released in July 2015. That document had no predecessor, nor does this supplement. WHO guidance on this species had been either included in recommendations regarding malaria generically, or added as virtual afterthoughts in documents taking aim principally at guiding strategy and tactics against *Plasmodium falciparum*. The *Technical Brief* thus acknowledges both the importance of *P. vivax* as a global health problem and the requirement to adopt tactics and strategies rationally suited to the singular features of this species. That consideration explains the voluminous character of this supplement—*P. vivax* is a more complicated parasite and clinical/public health problem than *P. falciparum*.

Complexities relevant to recommendations in the *Technical Brief* are explained here in detail, with extensive referencing pointing out the relevant evidence. This supplement may thus be viewed as a resource for technical people engaging in the difficult business of researching or controlling and eliminating this pernicious and stubborn malaria problem.

## A Singular Problem

Many of the contributors to the *Technical Brief* are represented in this supplement as authors. Most have a great deal of experience and expertise with *P. vivax*. These scientists and clinicians learned to cope with the complexity of this infection, either as a scientific, clinical, or public health problem, and this supplement covers all of these facets. Each paper may be classified under two broad categories: thematic review or country landscape. The thematic reviews each cover some broad topic of relevance to the control and elimination of *P. vivax*. The nations represented by the country landscapes offer a constellation of settings reflecting the diverse arrays of challenges posed by a globally distributed infection. These papers emphasize the need to adopt strategies suited to the character of the problem locally and the capacities of those attacking it.

In sum, this supplement describes the many unique features of *P. vivax* that bear directly upon its control and elimination. *Plasmodium vivax* causes two distinct infection syndromes—one actively proliferative and the other dormant or latent—each of distinct epidemiology and therapeutic requirements. Falciparum malaria has no latency. Latent *P. vivax* malaria cannot be diagnosed and relapse behaviors vary tremendously by geography. In many tropical zones, acute attacks originating from latent parasites dominate over sporozoite-borne primary attacks. The only known therapies effective against latent vivax malaria, 8-aminoquinoline drugs, invariably provoke acute hemolytic anemia in patients deficient in glucose-6-phosphate dehydrogenase (G6PD)—a highly polymorphic inherited disorder affecting an average of 8% of residents of malaria-endemic nations. The single low dose of primaquine against gametocytes of *P. falciparum* does not threaten the G6PD deficient. Waves of infectious gametocytes appear in peripheral blood before the onset of acute attacks of *P. vivax*, whereas those of *P. falciparum* appear a few days afterward. The parasitemia of patent vivax malaria is typically an order of magnitude lower than falciparum malaria, causing larger proportions of parasitemias to fall below diagnostics detection thresholds. Although clinically threatening falciparum malaria typically occurs with relatively high parasitemia, vivax malaria patients may exhibit very low parasitemias, and yet become severely ill. This may be a consequence of the bulk of *P. vivax* biomass occurring beyond vascular sinuses in tissues of the marrow and spleen, whereas *P. falciparum* biomass is impounded within vascular sinuses. These fundamental distinctions between the two dominant human malarias demand consideration in strategies of scientific research, clinical medicine, and public health aimed at combatting them.

Among the many important technical messages communicated within this supplement, a common theme resounds: controlling and eliminating *P. vivax* will require conceiving, optimizing, and validating tools and strategies aimed specifically at it. Tools developed and deployed for *P. falciparum* achieved extraordinary gains, but these alone will not suffice for *P. vivax*. The evidence, analysis, rationale, and strategic thinking captured in this supplement and summarized in the *Technical Brief* explain the insufficiency of the mainstay commodities of conventional malaria control; rapid diagnostic tests, artemisinin-based combined therapies, and long-lasting insecticide-treated nets. In addition to those conventional tools, the *P. vivax* control and elimination toolbox should also include:

1. Practical point-of-care G6PD deficiency diagnostics allowing far broader access to safe primaquine therapy or with tafenoquine, a related single dose hypnozoitocide in advanced development
2. More sensitive point-of-care diagnostics for detecting intrinsically lower parasitemias, including subpatent and asymptomatic infections
3. Validated strategies for relapse prevention in those lacking access to 8-aminoquinoline hypnozoitocides (pregnant women, young infants, G6PD deficients, and G6PD unknowns)
4. Clinical care algorithms acknowledging risk of severe and threatening syndromes despite seemingly nonthreatening levels of parasitemia
5. Interventions of proven efficacy to minimize human contact with often zoophilic and exophilic anopheline species of great diversity

This listing gives an appreciation of the character of the broad problem and what is needed to control and eliminate it. Intervening against the hypnozoite reservoir is key, and primaquine toxicity in G6PD-deficient patients directly obstructs doing so. Most acute attacks of *P. vivax* in endemic areas originate from hypnozoites, and unless that reservoir is aggressively and safely attacked, elimination of transmission may be an unrealistic goal. But this obstacle also presents a great opportunity—the historically unmolested hypnozoite reservoir in endemic communities may represent a key vulnerability in this species. If > 80% of attacks derive from it, surely killing that reservoir may quickly push this species to unsustainable transmission and endemic collapse as models consistently suggest. Except for the systematic and highly effective assault on endemic *P. vivax* in the former Soviet Union during the 1950s and 1960s using the 8-aminoquinoline called quinocide,^2^ humanity has yet to seriously assault the hypnozoite reservoir as a means of reducing or breaking endemic transmission.

## Implementing an Attack on Vivax Malaria

The task of controlling and eliminating *P. vivax* falls on the shoulders of the national malaria control programs (NMCPs) and their partners, local and international. They stand at the interface between enabling science and technology and those infected and in need of its benefits. The weight and complexity of the *P. vivax* problem and its historic neglect detailed in this supplement should not be misconstrued as imposing an inability of the NMCPs to immediately act decisively—they can dramatically improve access to and effectiveness of hypnozoitocidal therapy, for example.

Successfully translating the measures recommended in the *Technical Brief* into policy and routinely implemented practice where endemic vivax malaria occurs requires specific and deliberate actions guided by rational strategy. Many of these will require further evidence derived from a defined research agenda for *P. vivax*, but one of the most important actions—hypnozoitocidal therapy with each diagnosis of the infection in patients eligible to safely receive it—may be implemented with roll out of point-of-care G6PD deficiency screening to the peripheries of care delivery. Although those diagnostics may be improved in the coming years, the evidence informing their necessity and safety suffices to impel immediate action on this key front of attack.

Mobilizing primaquine may require understanding and acknowledging the basis of its six decades of availability, but lack of accessibility to the vast majority of patients in need of it. We have had the capacity to attack the hypnozoite reservoir, but failed to do so as a result of not understanding its epidemiology or clinical and public health consequences. Overcoming the toxicity problem with primaquine in endemic communities failed to register as a problem in need of a solution until just a few years ago. Entrepreneurial science and technology responded and provided a solution—G6PD point-of-care diagnostics suited to use at the peripheries of care delivery in the rural tropics. Now that we can do so safely, we need only grasp the necessity of attacking endemic latent *P. vivax*, doing so with vigor and with a vision of the tremendous health dividends very likely to follow.

## Elimination

The World Health Assembly in May 2015 adopted *The Global Technical Strategy for Malaria 2016–2030* (GTS) expressing the path forward to, “a world free of malaria.”^3^ GTS emphasizes acceleration of control in moving toward elimination. Effective control thus rationally precedes deliberate measures at elimination. Conventional malaria control has historically taken aim principally at *P. falciparum*, and even aggressive implementation has few lasting impacts upon endemic *P. vivax*. Poor diagnostics, therapies not impacting the hypnozoite reservoir, and the multiple recurrent attacks occurring without the involvement of a mosquito account for the tenacity of *P. vivax* in the face of control measures not rationally suited to it. Until nations accelerate the control of endemic *P. vivax* with appropriate strategies and tactics, progression to an elimination footing may remain elusive.

No single approach against *P. vivax*, including attacking the hypnozoite reservoir, will eliminate it. This resilient species requires multiple fronts of attack that include the asymptomatic and subpatent reservoirs along with measures aimed at reducing human contact with its plethora of mosquito vector species in enormously diverse ecologies of transmission. The evidence gathered in this supplement aims to guide the necessary research and optimized implementation of strategies that, collectively, may ultimately rid humanity of this pernicious malaria problem.
